# Intraosseous access in resuscitation

**DOI:** 10.1186/s13054-025-05651-w

**Published:** 2025-10-21

**Authors:** Renxian Xie, Lifeng Xiao

**Affiliations:** 1 Department of Radiation Oncology, Cancer Hospital of Shantou University Medical College, Shantou, People’s Republic of China; 2 Department of Emergency, Cancer Hospital of Shantou University Medical College, 7 Raoping Road, Shantou, 515031 Guangdong People’s Republic of China

Intraosseous (IO) access has gained traction as a primary vascular access method during resuscitation. Recent prehospital clinical trials, including the PARAMEDIC-3 [1] trial and VICTOR study [2], demonstrate no significant difference in 30-day survival between IO and intravenous (IV) access in adult out-of-hospital cardiac arrest (OHCA) patients [3]. This evidence has prompted a trend toward prioritizing IO access during OHCA, with some practitioners continuing its use post-return of spontaneous circulation (ROSC) during in-hospital care. However, only one study has evaluated IO access for in-hospital cardiac arrest (IHCA) [4], raising questions about its broad applicability in IHCA scenarios.

While IO access offers higher first-attempt success rates and rapid fluid delivery in hypovolemic shock patients with collapsed peripheral veins [5], its time advantage is unsubstantiated for non-traumatic arrests. The PARAMEDIC-3 trial revealed identical median access times for IO and IV accesses at 12 min, with comparable medication administration times [1]. Similarly, the VICTOR trial reported equivalent median access establishment times [2]. Within hospital settings, where IV or central venous catheter (CVC) placement is more feasible, the theoretical time advantage of IO access further diminishes.

Pharmacokinetic concerns also challenge IO access superiority during cardiopulmonary resuscitation (CPR). Epinephrine must rapidly reach the central circulation and achieve therapeutic concentrations to be effective. Previous research demonstrates that the route of epinephrine administration critically determines peak concentration and time to peak concentration in the central circulation [6]. When administered via the IO access, epinephrine must transit through bone marrow before entering systemic circulation. This process may cause partial drug deposition within the medullary cavity, reducing epinephrine concentration in peripheral circulation—an effect particularly pronounced with the initial bolus dose. Furthermore, the reticulated sinusoidal network within the marrow space delays drug entry into circulation, as illustrated in Fig. 1.

Evidence indicates IO access delivery prolongs time-to-peak concentration by 1.4–2.5-fold compared to IV access. The consequent reduction in peak concentration in the central circulation has prompted proposals for higher IO epinephrine dosing [7, 8]. Epinephrine’s dual mechanisms—enhancing coronary perfusion pressure and directly stimulating cardiomyocytes—make rapid cardiac delivery imperative. However, cardiopulmonary resuscitation interferes with this process: extrathoracic compressions increase intrathoracic pressure, which impedes venous return and delays drug delivery. Evidence indicates that intraosseous access delays time to ROSC in both IHCA and OHCA settings [9, 10]. Furthermore, predominant lower extremity IO access placement increases drug transit distance to the heart, compounding delays. Delayed epinephrine administration significantly reduces the ROSC rate and results in a poorer neurologic prognosis for the patient, an effect that is most pronounced within the first 10 min [11]. In the IHCA setting, where epinephrine administration can be accelerated, there is no clear advantage to IO access-although there are no randomized controlled trials to confirm this.

Practical limitations warrant consideration. IO access requires specialized equipment and pressurized infusion systems to approach CVC flow rates. Prolonged retention beyond 24 h increases infection risk. Significant infusion pain necessitates lidocaine co-administration in conscious patients, introducing potential arrhythmogenic effects from this adjunct medication. The clinical implications of such pharmacological interactions remain uncertain.

Current evidence indicates no significant difference in survival or neurological outcomes between IO and IV access in patients with OHCA [12]. Furthermore, there is insufficient evidence to support IO access as the primary vascular pathway for IHCA. Existing evidence and pharmacokinetic principles do not support its routine prioritization in in-hospital settings.

**Fig. 1:**
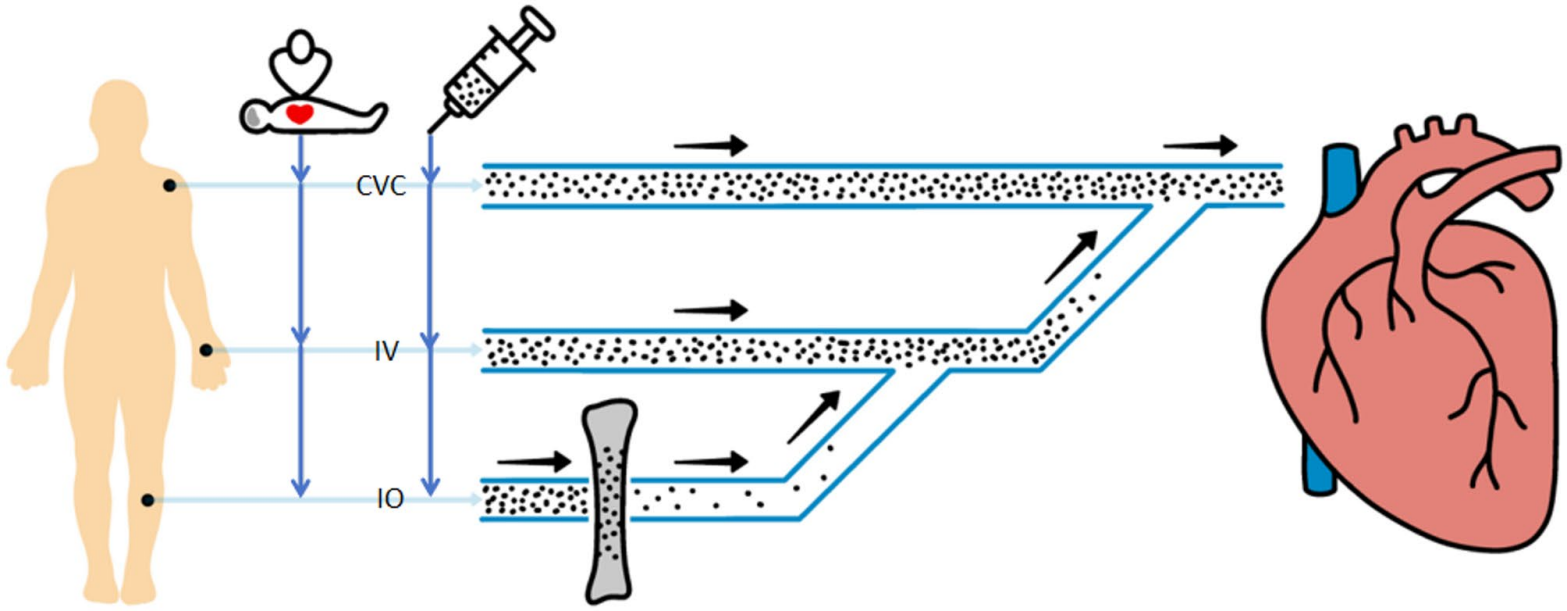
Schematic representation of the pharmacokinetic properties of the different vascular pathways during resuscitation

## Data Availability

No datasets were generated or analysed during the current study.
